# The Adjuvant-Phase Dilemma After Neoadjuvant Chemoimmunotherapy in Resectable NSCLC: Evidence and Response-Guided Strategies

**DOI:** 10.3390/cancers18182933

**Published:** 2026-09-10

**Authors:** Jingyuan Fan, Yichao Han, Runsen Jin, Hecheng Li

**Affiliations:** Department of Thoracic Surgery, Ruijin Hospital, School of Medicine, Shanghai Jiao Tong University, Shanghai 200025, China; caroline_fanjy@sjtu.edu.cn (J.F.); hyc01k44@rjh.com.cn (Y.H.)

**Keywords:** non-small-cell lung cancer, neoadjuvant therapy, perioperative therapy, immune checkpoint inhibitors, biomarkers

## Abstract

**Background/Objectives**: Perioperative chemoimmunotherapy has become an important curative-intent strategy for selected patients with resectable non-small cell lung cancer (NSCLC). However, most pivotal trials evaluate neoadjuvant therapy, surgery, and postoperative immune checkpoint inhibitor (ICI) treatment as an integrated regimen. Whether continued postoperative immunotherapy provides independent incremental benefit for all patients after effective neoadjuvant chemoimmunotherapy and complete resection remains unresolved. This review focuses on the adjuvant-phase dilemma and discusses how postoperative treatment may be refined according to response and residual risk. **Methods**: We performed a narrative review of major neoadjuvant and perioperative chemoimmunotherapy trials, indirect comparative analyses, pathological-response studies, circulating tumor DNA (ctDNA)-based molecular residual disease (MRD) evidence, immune biomarker studies, and emerging data in driver-positive and real-world populations. Particular attention was given to evidence informing postoperative continuation, de-escalation, or intensification after neoadjuvant chemoimmunotherapy. **Results**: Current phase III perioperative trials demonstrate clinically meaningful activity but do not isolate the independent contribution of the adjuvant ICI phase. Pathological response provides the most accessible postoperative risk signal: pathologic complete response identifies the deepest-response group, major pathologic response represents an intermediate state, and non-major pathologic response or persistent nodal disease suggests higher relapse risk. ctDNA-based MRD offers dynamic risk refinement and may help identify patients with residual systemic disease, although prospective validation is required before it can guide routine treatment omission or escalation. Programmed death-ligand 1 (PD-L1), tumor mutational burden, tertiary lymphoid structures, B-cell signatures, radiomics, and pathomics may provide complementary information but are not sufficient as standalone decision tools. Driver-positive disease requires molecularly stratified perioperative strategies rather than unselected extrapolation from epidermal growth factor receptor (EGFR)/anaplastic lymphoma kinase (ALK)-negative trials. **Conclusions**: The key question in resectable NSCLC is shifting from whether perioperative immunotherapy is active to which patients truly require postoperative immunotherapy. Future trials should prospectively test response-guided strategies integrating pathological response, ctDNA-based MRD, immune contexture, baseline risk, and treatment feasibility.

## Simple Summary

Perioperative immunotherapy, which includes treatment before and after surgery, has improved outcomes for some patients with resectable non-small cell lung cancer. However, current clinical trials usually test the entire treatment plan rather than the separate value of continuing immunotherapy after surgery. This raises an important question: does every patient need postoperative immunotherapy, or can treatment be adjusted according to their response and remaining risk? This review discusses how pathological response in the surgically removed tumor and lymph nodes, baseline clinical features of the tumor and its immune environment, postoperative monitoring of molecular residual disease, surgical recovery, and treatment feasibility may together inform future postoperative treatment strategies. However, none of these factors has yet been prospectively validated to independently determine postoperative treatment. We propose that future studies should evaluate whether patients with deep responses can safely receive less postoperative treatment, while patients with residual disease or high relapse risk may benefit from continued or intensified therapy.

## 1. Introduction

Lung cancer remains the leading cause of cancer-related mortality worldwide, accounting for over 1.8 million deaths annually. Non-small cell lung cancer (NSCLC) accounts for 80–85% of cases, encompassing subtypes such as adenocarcinoma, squamous cell carcinoma, and large cell carcinoma [1]. The treatment of NSCLC is primarily guided by clinical staging. While early-stage disease is often amenable to surgical resection, locally advanced NSCLC, which accounts for 30% of newly diagnosed cases, poses significant challenges. Tumor burden and mediastinal lymph node involvement impede complete (R0) resection, while the high recurrence rate following R0 resection underscores an urgent need for effective systemic therapies [2].

In response to these challenges, immunotherapy has emerged as a transformative strategy in oncology. Immune checkpoint inhibitors (ICIs) targeting programmed cell death protein 1 (PD-1)/PD-L1 and cytotoxic T-lymphocyte-associated protein 4 (CTLA-4) have significantly improved outcomes in advanced NSCLC and are now being explored in earlier stages [3]. However, although multiple trials have established the efficacy of perioperative ICI-based regimens in resectable NSCLC, they primarily evaluate perioperative treatment as an integrated strategy. Consequently, whether continued postoperative immunotherapy provides independent incremental benefit after effective neoadjuvant chemoimmunotherapy and complete resection remains unresolved. Against this background, this review focuses on the adjuvant-phase dilemma after neoadjuvant chemoimmunotherapy in resectable NSCLC and discusses how pathological response, molecular residual disease (MRD) status, treatment burden, and emerging biomarkers may inform future response-guided postoperative strategies.

## 2. Literature Search and Evidence Selection

A focused literature search was performed using PubMed, Embase, Google Scholar, and ClinicalTrials.gov for English-language studies relevant to perioperative systemic therapy in resectable NSCLC from January 2015 to August 2026. Search terms combined concepts related to resectable NSCLC; neoadjuvant, perioperative, and adjuvant systemic therapy; pathological response and postoperative risk stratification; molecular residual disease and other biomarkers; and oncogene-driven targeted therapy. Representative terms included “NSCLC”, “resectable NSCLC”, “neoadjuvant”, “perioperative”, “adjuvant”, “immunotherapy”, “chemoimmunotherapy”, “pathological response”, “molecular residual disease”, “biomarker”, and “targeted therapy”.

Titles and abstracts were screened for relevance, and potentially relevant publications were subsequently reviewed in full text. Evidence was selected according to its relevance to the central clinical questions of this review, particularly postoperative treatment decision-making after neoadjuvant chemoimmunotherapy. Priority was given to phase III randomized trials and their updated analyses, followed by prospective studies, meta-analyses, and clinically relevant retrospective cohorts addressing pathological response, MRD, biomarkers, targeted therapy, surgical feasibility, or postoperative treatment strategies. Relevant ongoing trials were also identified to characterize emerging therapeutic strategies and unresolved research questions. When multiple reports from the same trial were available, the most recent mature analysis was prioritized, while earlier or secondary reports were retained when they provided unique outcomes relevant to this review. Literature identification and relevance assessment were conducted by two authors, with disagreements resolved through discussion and consensus.

## 3. Overview of Neoadjuvant and Perioperative Treatment Regimens: Evidence and Remaining Gaps

### 3.1. Neoadjuvant and Perioperative Chemoimmunotherapy Regimens

Extensive clinical investigations have evaluated immune checkpoint inhibitors in the curative-intent management of resectable NSCLC. These studies can be broadly divided into neoadjuvant chemoimmunotherapy, in which ICIs are administered before surgery [4], and perioperative chemoimmunotherapy, in which postoperative ICI treatment is continued after neoadjuvant chemoimmunotherapy and resection [5].

Neoadjuvant immunotherapy aims to enhance immune activation within the tumor microenvironment before surgery, potentially reducing micrometastatic spread and promoting systemic antitumor memory [4]. CheckMate 816 established nivolumab plus platinum-doublet chemotherapy as a landmark neoadjuvant regimen, improving pathological response and event-free survival (EFS) compared with chemotherapy alone. Other neoadjuvant studies, including TD-FOREKNOW and NEOSTAR, further supported the feasibility and activity of preoperative ICI-based strategies.

Perioperative chemoimmunotherapy extends this concept by adding a postoperative adjuvant ICI phase after surgery [6]. Trials such as KEYNOTE-671, AEGEAN, CheckMate 77T, Neotorch, NADIM II, and RATIONALE-315 have evaluated this integrated approach, aiming to combine preoperative immune priming with sustained postoperative systemic control. Several of these regimens have demonstrated significant improvements in pathological response and EFS, and some have now reported more mature overall survival (OS) follow-up. However, the absence of direct comparative data within a unified clinical framework continues to fuel debates between neoadjuvant and perioperative regimens, necessitating a head-to-head comparison in the future [7].

### 3.2. Patient Selection, Efficacy, and Safety Considerations

Patient selection differed across major trials, which limits direct comparison between neoadjuvant-only and perioperative strategies. Most studies focused on high-risk resectable NSCLC, typically stage II–III disease, tumors ≥4 cm, or node-positive disease, while patients with known EGFR mutations or ALK rearrangements were generally excluded. Subgroup analyses from individual trials suggested that perioperative regimens may provide clinically meaningful benefit in higher-risk populations, including patients with PD-L1-negative disease, stage III disease, squamous histology, or nodal involvement [4,8,9]. However, these findings remain exploratory and should not be used to infer that all patients with such features require postoperative immunotherapy.

Overall efficacy was encouraging across both treatment paradigms, but the available data should be interpreted as an evidence landscape rather than a direct comparison. In the updated trial table, neoadjuvant-only regimens reported pathologic complete response (pCR) rates of approximately 16.2–32.6% and major pathologic response (MPR) rates of 32.1–65.1%, whereas perioperative regimens reported pCR rates of 17.2–41.0% and MPR rates of 30.2–62.0% (Table 1) [10]. Among representative phase III trials, KEYNOTE-671 reported a median EFS of 57.1 months, with updated 5-year EFS and OS rates of 49.9% and 64.6%, respectively; CheckMate 816 reported a median EFS of 59.6 months, with updated 5-year EFS and OS rates of 49.2% and 65.4%, respectively [4,9,11,12]. Other neoadjuvant and perioperative studies further support the activity of ICI-based strategies, but their outcomes are best summarized as ranges because of differences in trial design, follow-up maturity, and postoperative treatment exposure.

Safety considerations should be interpreted in the context of treatment duration. Grade ≥ 3 treatment-related adverse event (TRAE) and immune-related adverse event (irAE) rates varied substantially across studies, reflecting differences in chemotherapy backbone, ICI agent, reporting methods, and treatment exposure. In general, neoadjuvant-only studies reported high-grade TRAE rates of approximately 10.0–45.0%, whereas perioperative studies with available data reported rates of approximately 32.0–78.4% (Table 1) [13]. Preliminary health-related quality of life (HRQoL) data from KEYNOTE-671 suggested generally preserved postoperative quality of life, although only selected domains showed statistically significant improvement and these findings remain insufficient to guide treatment selection [14,15]. Taken together, current efficacy and safety data support the feasibility of both neoadjuvant and perioperative chemoimmunotherapy, but they do not resolve whether postoperative immunotherapy should be continued universally after surgery (Table 1).

### 3.3. The Evidence Gap: Does the Adjuvant Phase Add Incremental Benefit?

In KEYNOTE-671, AEGEAN, CheckMate 77T, Neotorch, and RATIONALE-315, patients were assigned to an integrated strategy consisting of neoadjuvant chemoimmunotherapy, surgery, and continued adjuvant ICI treatment. Therefore, improvements in EFS or OS reflect the benefit of the entire perioperative regimen, rather than the isolated effect of postoperative immunotherapy. Since patients were not re-randomized after surgery to adjuvant ICI under the same neoadjuvant backbone, these trials cannot determine whether all patients truly derive incremental benefit from continued immunotherapy after complete resection.

Recent indirect evidence has reinforced this uncertainty rather than resolved it. In a network meta-analysis comparing seven perioperative ICI strategies, the researchers found no clear gain in EFS (hazard ratio [HR] = 0.97, 95% confidence interval [CI]: 0.67 to 1.41; *p* = 0.87) or OS (HR = 1.17, 95% CI: 0.59 to 2.31, *p*  = 0.65) over neoadjuvant chemoimmunotherapy [16]. A second network meta-analysis reported a similar result for EFS (HR = 0.78, 95% CI: 0.37 to 1.62; *p* = 0.51). However, they found that in subgroups of patients with PD-L1-negative disease or Eastern Cooperative Oncology Group performance status (ECOG PS) ≥ 1, perioperative treatment was associated with significantly improved EFS compared with neoadjuvant treatment, albeit at the expense of increased grade ≥3 toxicity [17]. But again, this evidence failed to isolate the specific contribution of the postoperative adjuvant component—a limitation shared by other meta-analyses as well [18,19]. Collectively, these studies suggest that regimen-level efficacy should not be equated with universal adjuvant-phase benefit.

This evidence gap shifts the clinical question from whether perioperative immunotherapy is active to which patients truly require the postoperative component. Pathological-response analyses provide a more clinically relevant way to frame this uncertainty. Since pathological response is routinely available after surgery and is consistently associated with long-term outcomes, it currently provides the most practical first clue for postoperative risk stratification. The next section therefore examines pCR, MPR, and non-MPR as potential decision nodes for de-escalation, continuation, or intensification after neoadjuvant chemoimmunotherapy.

## 4. Pathological Response as an Emerging Clue for Response-Guided Postoperative Strategies

Pathological response is currently the most accessible postoperative signal after neoadjuvant chemoimmunotherapy. Unlike baseline biomarkers, it reflects the integrated effects of tumor biology, chemotherapy sensitivity, immune activation, and host response after treatment. In the adjuvant-phase dilemma, its value is not to provide an immediate rule for omitting postoperative therapy, but to stratify residual relapse risk after surgery and guide future de-escalation or escalation trials.

### 4.1. pCR and MPR as Favorable Prognostic Markers

pCR and MPR are consistently associated with favorable long-term outcomes after neoadjuvant chemoimmunotherapy in resectable NSCLC, as demonstrated across landmark trials including CheckMate 816, KEYNOTE-671, AEGEAN, NADIM II, Neotorch, and RATIONALE-315 [4,8,15,20,21]. In KEYNOTE-671, a post-hoc analysis based on percent residual viable tumor (%RVT) further confirmed that lower %RVT correlates with improved EFS, reinforcing pathological response as a clinically meaningful postoperative readout [22].

Meta-analytic evidence substantiates this association [23]. A reconstructed individual-patient-data meta-analysis reported strong EFS associations for both pCR versus non-pCR (HR = 0.13) and MPR versus non-MPR (HR = 0.18), with corresponding 24-month EFS rates of approximately 94% versus 54%, and 88% versus 48%, respectively [24]. A retrospective cohort evaluating postoperative adjuvant ICI therapy after neoadjuvant immunotherapy further supported that the postoperative therapy could significantly improve EFS in resectable NSCLC patients, especially in those without pCR or MPR (*p* = 0.004). However, its effect on OS remains uncertain [25].

Despite both being favorable, pCR and MPR should not be treated as interchangeable. pCR represents the deepest measurable response, with 0% residual viable tumor (RVT) and minimal relapse risk, whereas MPR is defined by ≤10% RVT and encompasses a more heterogeneous group among patients with residual viable tumor. This distinction may have therapeutic implications: a recent meta-analysis comparing neoadjuvant and perioperative ICI-based regimens found that among patients achieving MPR, perioperative treatment was associated with improved EFS (*p* = 0.038), whereas no such benefit was observed in pCR achievers (*p* = 0.408) [26]. These findings raise the possibility that patients achieving MPR may derive greater incremental benefit from continued postoperative immunotherapy than those achieving pCR. Accordingly, MPR should be considered separately from pCR when designing and evaluating future response-guided postoperative strategies, rather than being grouped together as a single favorable-response category.

### 4.2. Non-MPR and Incomplete Responders as Candidates for Postoperative Intensification

Patients with incomplete pathological response occupy the higher-risk end of the postoperative spectrum. Residual viable tumor greater than 10%, persistent nodal disease, or postoperative molecular residual disease may indicate incomplete eradication of resistant tumor clones or insufficient immune-mediated tumor clearance. Reconstructed individual patient data meta-analysis has confirmed a prognostic gradient from pCR (good) through MPR 1–10% (intermediate) to non-MPR (poor) [27]. Real-world evidence also suggested an association between additional adjuvant ICI therapy and improved EFS among patients who did not achieve pCR or MPR, whereas no clear association was observed among patients achieving deeper pathological responses [25,28]. A multicenter retrospective cohort further proposed a treatment pattern–pathological response framework for postoperative decision-making, suggesting that non-MPR may identify a population warranting prospective evaluation of postoperative intensification [29].

Nonetheless, most available studies mainly stratified postoperative risk according to primary-tumor response, while primary-tumor regression alone does not fully characterize postoperative pathological risk. Primary-tumor response and postoperative nodal status represent complementary but non-equivalent pathological dimensions. Favorable regression within the primary tumor does not necessarily imply nodal clearance. Recent multicenter retrospective studies showed that persistent ypN-positive disease after neoadjuvant chemoimmunotherapy was associated with substantially poorer survival than ypN0, and that patients with MPR but persistent nodal disease had worse outcomes than those with MPR and ypN0 [30,31]. These findings indicate that postoperative nodal status provides prognostic information beyond residual viable tumor in the primary lesion. A recent multicenter cohort of 363 patients further suggested that combining MPR with ypN status may provide more granular postoperative stratification, with an adjuvant-immunotherapy benefit signal observed predominantly in the non-MPR/ypN-positive subgroup [32]. Considering that the postoperative treatment was not randomized, this finding remains hypothesis-generating rather than establishing MPR–ypN classification as a predictive treatment-selection biomarker.

Taken together, residual primary-tumor burden and persistent ypN disease identify patients with higher postoperative relapse risk, particularly when both features coexist. However, these pathological features do not by themselves establish which postoperative treatment strategy is optimal. The NeoTAP01 4-year follow-up further illustrates this uncertainty, showing that 75% of recurrences among non-pCR patients occurred within one year after surgery despite adjuvant treatment [33]. Thus, non-MPR and persistent ypN disease may help refine future response-guided postoperative strategies, but should not yet dictate routine treatment escalation.

### 4.3. Ongoing Trials and Response-Based Postoperative Strategies

The limitations of current retrospective and indirect evidence highlight the need for component-specific trials that directly determine whether postoperative ICI provides additional benefit after neoadjuvant chemoimmunotherapy and surgery. ADOPT-lung is the first randomized phase III trial specifically designed to address this question in resectable NSCLC. Following neoadjuvant platinum-doublet chemotherapy plus durvalumab and surgery, eligible patients are 1:1 randomized to adjuvant durvalumab or observation, with disease-free survival (DFS) in the non-pCR population as the primary endpoint [34]. Melanoma provides a related, although not identical, precedent for response-adapted postoperative treatment. In NADINA, neoadjuvant ipilimumab plus nivolumab followed by surgery and response-driven adjuvant therapy improved event-free survival compared with surgery followed by adjuvant nivolumab; within the neoadjuvant group, postoperative systemic therapy was reserved for patients with a pathological partial response or non-response [35]. The PRADO trial similarly demonstrated the feasibility of omitting both therapeutic lymph-node dissection and adjuvant therapy in patients achieving MPR after neoadjuvant ipilimumab plus nivolumab, while directing more intensive treatment toward poorer responders [36]. As no completed randomized NSCLC trial has yet reported the isolated contribution of postoperative ICI continuation after neoadjuvant chemoimmunotherapy, the results of ADOPT-lung and future trials incorporating more granular pathological stratification are highly anticipated. Future studies may further improve such response-adapted designs by incorporating postoperative nodal status alongside primary-tumor response rather than relying on pCR or MPR alone.

Overall, pathological response should be viewed as a prognostic and risk-stratification signal rather than a stand-alone treatment rule. A more informative framework is to interpret primary-tumor response together with postoperative nodal status: deep primary-tumor response with nodal clearance represents the most favorable pathological state and an attractive population for prospective de-escalation trials; MPR requires further refinement according to nodal status and other residual-risk features; and non-MPR or persistent ypN disease indicates higher postoperative risk, particularly when both are present.

## 5. From Pathological Response to Integrated Postoperative Strategies

Pathological response alone cannot fully capture the impact of baseline disease aggressiveness, residual systemic disease, or the feasibility of continued treatment. Therefore, a more clinically meaningful response-guided strategy should extend beyond the pCR/MPR/non-MPR classification and integrate complementary clinical, immunological, and molecular information.

### 5.1. Biomarkers for Baseline Risk Refinement

Baseline stage, nodal burden, tumor extent, ECOG performance status, and molecular characteristics establish the pretreatment probability of recurrence and provide essential context for interpreting postoperative response. In a network meta-analysis, perioperative treatment was associated with significantly improved EFS compared with neoadjuvant treatment among patients with ECOG PS ≥ 1, albeit at the expense of increased grade ≥3 toxicity [17]. Among tumor-associated biomarkers, PD-L1 expression has shown predictive or prognostic associations in several perioperative trials, including AEGEAN and KEYNOTE-671, although inconsistent findings in Neotorch and NADIM II limited its universal application [4,8,37,38,39,40]. Similarly, tumor mutational burden (TMB) has been linked to pathological response in some neoadjuvant studies, but its ability to identify patients who specifically require or can safely omit postoperative immunotherapy remains uncertain [41,42,43,44]. Thus, while clinical risk factors, PD-L1, and TMB may inform the baseline probability of recurrence or treatment benefit, none is sufficiently reliable to override pathological response or independently dictate adjuvant therapy.

Immune-contexture biomarkers, such as tertiary lymphoid structures (TLS), B-cell signatures, and B-cell receptor (BCR) repertoire characteristics, may provide a biologically distinct and second layer of risk refinement. A meta-analysis of 17 studies including 4291 lung cancer patients demonstrated that high TLS density was associated with improved OS (HR = 0.66, 95% CI: 0.50–0.88), with especially pronounced effects in Asian subgroups, suggesting its potential as a predictive marker for immunotherapy response and as contextual evidence for risk stratification [45]. Emerging evidence also suggests that BCR repertoire characteristics are associated with pathological response and may offer information complementary to PD-L1 and TMB [46]. Meanwhile, radiomic and pathomic approaches may further capture intratumoral heterogeneity and treatment-induced remodeling, although their predictive value for postoperative treatment selection requires prospective validation [47,48].

Taken together, these clinical, molecular, and immune-contexture features provide complementary modifiers of pathological response rather than independent treatment-selection biomarkers. Most of them remain relatively static or indirectly associated with relapse risk. A more direct postoperative strategy therefore requires a dynamic, longitudinal biomarker capable of detecting whether residual systemic disease persists after neoadjuvant treatment and surgery.

### 5.2. ctDNA-Based MRD for Dynamic Postoperative Risk Assessment

Whereas clinical characteristics and tissue biomarkers primarily define baseline or contextual risk, circulating tumor DNA (ctDNA)-based MRD provides a dynamic assessment of how that risk evolves during treatment [49]. ctDNA consists of small fragments of tumor-derived DNA detectable in blood. ctDNA-based MRD refers to molecular evidence of residual malignant disease after treatment, even when no disease is clinically or radiographically apparent. It can be assessed using different assay platforms and at predefined postoperative landmarks or longitudinally throughout treatment and surveillance [50]. This temporal dimension distinguishes MRD from baseline PD-L1 expression or TMB and makes it particularly relevant for patients with similar pathological responses but potentially different risks of recurrence.

For the adjuvant-phase dilemma, accumulating evidence suggests that postoperative ctDNA may identify patients more likely to benefit from continued therapy. In the LUNGCA-1 prospective cohort, ctDNA-detected MRD positivity at postoperative day 3 and/or 1 month strongly predicted relapse and outperformed conventional clinicopathological variables including TNM stage. Notably, adjuvant therapy was associated with improved recurrence-free survival (RFS) in MRD-positive patients, whereas no such association was observed in MRD-negative patients after adjustment [51]. A separate study evaluating MRD-guided adjuvant decisions reached a similar conclusion: landmark MRD positivity at one month after R0 resection was associated with markedly shorter DFS, while MRD-negative patients derived limited apparent benefit from adjuvant strategies [52]. Complementary studies showed that sustained MRD negativity defined a very-low-risk population, whereas residual ctDNA predicted relapse months before imaging, supporting ctDNA-positive patients as candidates for intensified surveillance, or postoperative intervention [53,54].

Despite these findings, MRD should not yet be used as a standalone criterion to omit or mandate adjuvant immunotherapy. Technical and biological barriers remain, including assay and platform variability, analytical sensitivity, low or absent ctDNA shedding, sampling schedules, and standardization [50]. Importantly, a negative MRD result does not necessarily indicate the complete absence of residual malignant disease. A recent meta-analysis reported higher sensitivity with longitudinal than landmark MRD assessment (0.74 vs. 0.50), highlighting the limitations of relying on a single postoperative measurement [55]. The optimal postoperative sampling schedule also remains undefined, and further standardization across assays and time points is required [56]. Future improvements in genomic, epigenetic, and longitudinal detection strategies may further enhance MRD sensitivity and accuracy [57,58]. Most importantly, MRD-guided postoperative immunotherapy has not yet been prospectively validated. Therefore, MRD is better viewed as a risk-refinement tool to be integrated with pathological response, TNM stage, and clinical risk—guiding future trial design and patient stratification rather than dictating current clinical decisions.

### 5.3. Surgical Course and Postoperative Treatment Feasibility

Surgery represents the critical transition between neoadjuvant treatment and the postoperative phase. Importantly, operative burden is not determined by neoadjuvant therapy alone. Baseline tumor location and extent, nodal involvement, anticipated extent of resection, and cardiopulmonary reserve may influence the planned surgical approach and expected physiological burden. Neoadjuvant treatment may modify this course through tumor regression, while treatment-related inflammation and fibrosis can increase technical complexity, particularly during hilar and nodal dissection. A recent systematic review and meta-analysis of 27 prospective trials involving 2691 patients reported low pooled rates of intraoperative complications and postoperative mortality, while postoperative complications occurred in approximately one quarter of patients. Minimally invasive surgery remained feasible in a substantial proportion of cases, although conversion to open surgery and pneumonectomy were not uncommon [59]. These findings highlight the heterogeneity in operative complexity and postoperative recovery.

For the adjuvant-phase dilemma, the more relevant consideration is how the actual surgical course and recovery affect subsequent treatment feasibility. Extensive resection, perioperative morbidity, or delayed cardiopulmonary and functional recovery may postpone or limit postoperative systemic therapy. This is reflected in a recent meta-analysis of prospective immunotherapy trials, in which 9.6% of patients did not initiate planned adjuvant therapy and 34.6% required treatment discontinuation or cycle reduction; among perioperative protocols, postoperative complications accounted for 6.4% of adjuvant-treatment omissions [60]. Systemic-treatment burden adds a further constraint: perioperative chemoimmunotherapy has been associated with a higher incidence of grade ≥3 treatment-related adverse events than neoadjuvant approaches [17]. Thus, the postoperative phase should not be viewed as a “free” therapeutic extension after surgery. Overall, postoperative treatment feasibility should incorporate surgical burden, perioperative morbidity, cardiopulmonary and functional recovery, cumulative treatment toxicity and patient preference, rather than systemic-treatment tolerance alone.

### 5.4. An Integrated Response-Guided Clinical Framework

Taken together, postoperative decision-making can be conceptualized across two interdependent dimensions: biological residual risk and treatment feasibility. Biological residual risk comprises three sequential domains: baseline clinical risk (stage, pretreatment nodal burden, ECOG PS, and PD-L1 expression), postoperative pathological status (primary-tumor response [pCR, MPR, or non-MPR] and ypN status), and dynamic molecular status (postoperative ctDNA/MRD). Baseline risk defines the pretreatment propensity for relapse; postoperative pathology captures the tumor’s actual response to neoadjuvant therapy at both the primary and nodal sites; and MRD provides a dynamic readout of residual systemic disease. These domains should be interpreted sequentially rather than averaged.

Within this framework, pCR with sustained postoperative MRD negativity and favorable baseline biology represents the most favorable residual-risk profile, whereas MPR with ypN0 requires further refinement according to residual viable tumor burden, baseline risk, and MRD status. Non-MPR, persistent ypN disease (particularly ypN2), positive MRD, or high baseline stage III burden indicate progressively less favorable residual-risk profiles. Importantly, none of these features, alone or in combination, has been prospectively validated as a definitive postoperative treatment-selection rule.

Treatment feasibility modifies how biological risk may be translated into future postoperative strategies. As discussed above, the surgical course and recovery should be considered together with treatment-related toxicity and patient preference. Patients with concordantly favorable biological features and adequate recovery may represent appropriate populations for prospective de-escalation or observation trials, whereas those with persistent pathological or molecular risk and adequate recovery may be prioritized for prospective continuation or intensification studies. Conversely, substantial perioperative morbidity or delayed recovery may constrain postoperative treatment even when biological residual risk remains high.

**Figure 1:**
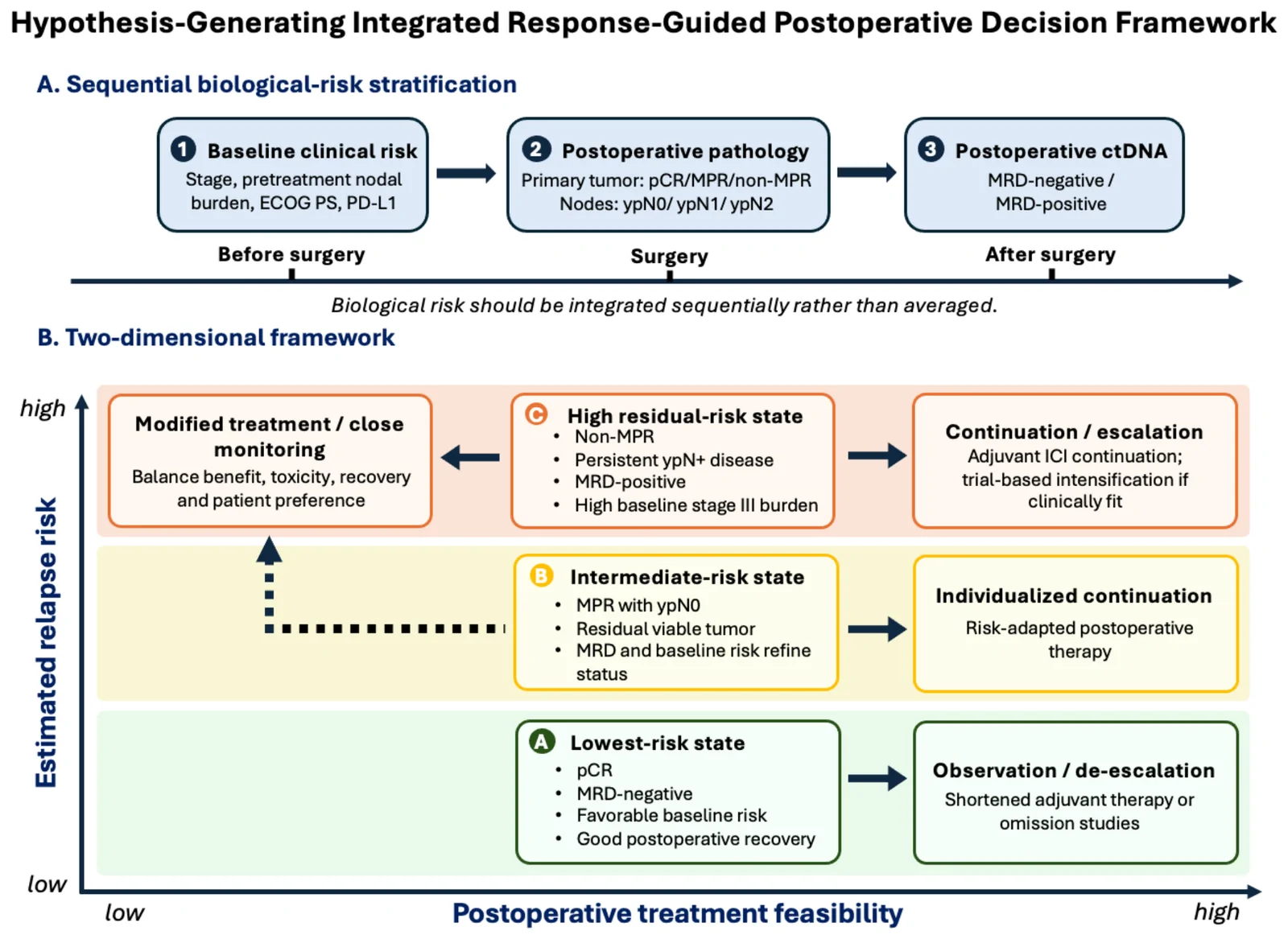
Hypothesis-Generating Integrated Response-Guided Postoperative Decision Framework. This framework is hypothesis-generating and has not been prospectively validated. It is intended to support multidisciplinary discussion and clinical-trial development and should not be interpreted as a recommendation to omit, continue, or escalate approved postoperative therapy in routine clinical practice. (**A**) Sequential biological-risk assessment integrates baseline clinical risk, postoperative pathological status, and postoperative ctDNA-based MRD. Postoperative pathology incorporates both primary-tumor response and ypN status, recognizing that favorable regression of the primary tumor does not necessarily imply nodal clearance. These domains should be interpreted sequentially rather than averaged. (**B**) The two-dimensional framework integrates estimated biological residual risk with postoperative treatment feasibility, which incorporates surgical burden, perioperative morbidity, cardiopulmonary and functional recovery, treatment-related toxicity, and patient preference. Concordantly favorable biological features and adequate recovery may define populations for prospective de-escalation or observation trials, whereas persistent pathological or molecular risk may identify populations for prospective continuation or intensification studies when clinically feasible. Abbreviations: ECOG PS, Eastern Cooperative Oncology Group performance status; ICI, immune checkpoint inhibitor; MPR, major pathological response; MRD, molecular residual disease; pCR, pathological complete response; PD-L1, programmed death-ligand 1; ypN, post-neoadjuvant pathological nodal status.

This framework is designed for multidisciplinary discussion and trial development rather than as a direct replacement for approved regimens (Figure 1). Its primary value is to define clinically meaningful populations for prospective testing of postoperative de-escalation, continuation, or intensification strategies.

## 6. Current Dilemmas and Future Directions

**Figure 2:**
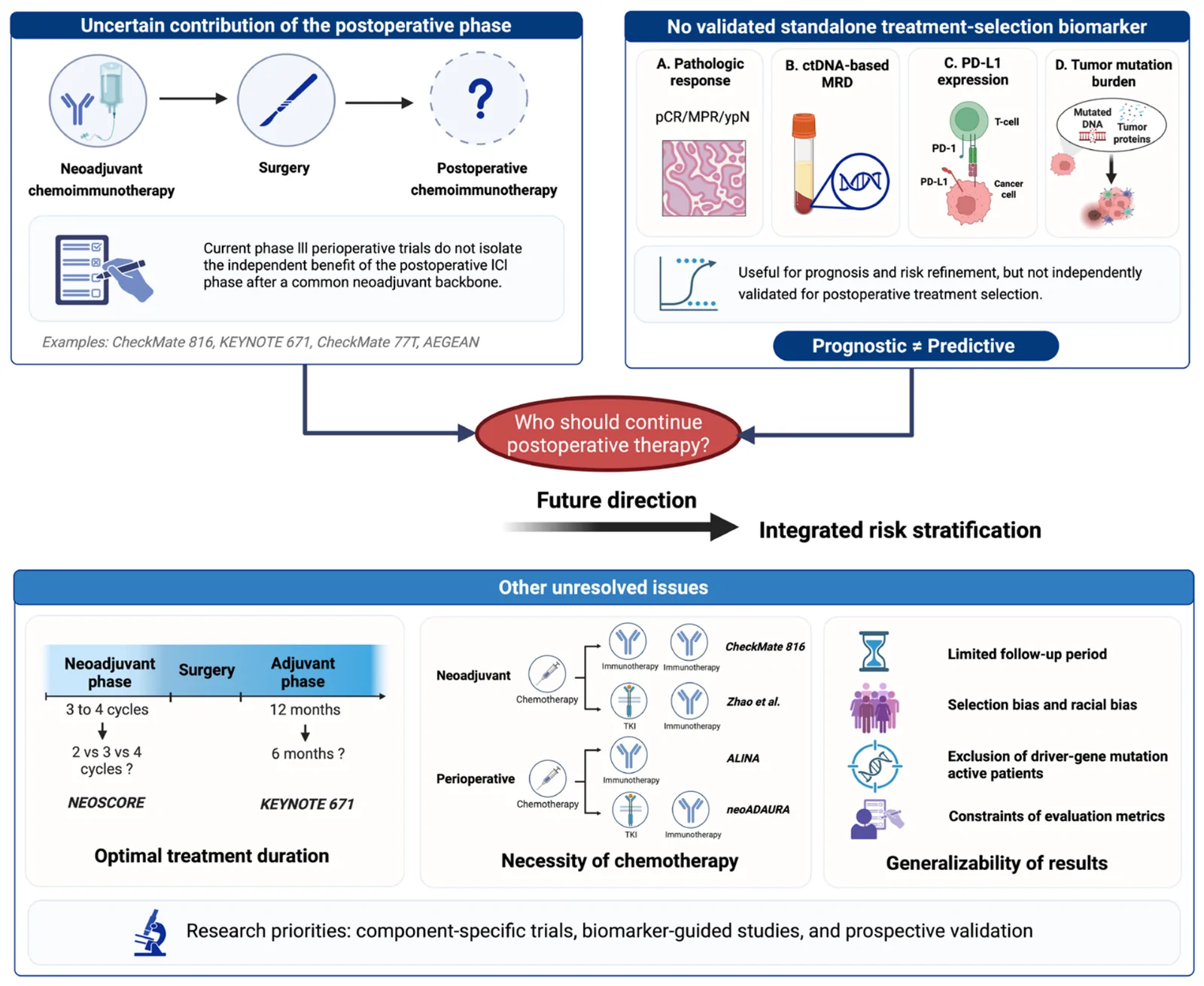
Current dilemmas and future directions in perioperative management of resectable NSCLC. The upper panels illustrate two key sources of uncertainty: the inability of current perioperative trial designs to isolate the independent contribution of postoperative ICI therapy, and the absence of a prospectively validated standalone biomarker for postoperative treatment selection. The central node highlights the clinical question of which patients should continue postoperative treatment, with integrated risk stratification presented as a potential future direction. The lower panels summarize additional areas requiring further investigation. This schematic is intended to illustrate research directions and should not be interpreted as a validated clinical decision algorithm [68]. Abbreviations: ctDNA, circulating tumor DNA; ICI, immune checkpoint inhibitor; MPR, major pathological response; MRD, molecular residual disease; pCR, pathological complete response; PD-1, programmed cell death 1; PD-L1, programmed death-ligand 1; ypN, post-neoadjuvant pathological nodal status.

Despite the established efficacy of perioperative chemoimmunotherapy as an integrated strategy, several clinically important dilemmas remain unresolved. First, current phase III trials do not isolate the incremental contribution of the postoperative ICI phase after a common neoadjuvant treatment and surgery. Second, although pathological response and ctDNA-based MRD provide valuable prognostic and risk-stratification information, no biomarker has been prospectively validated as a standalone criterion for postoperative treatment selection. Third, the optimal duration and intensity of postoperative therapy remain uncertain, and any potential benefit must be balanced against cumulative toxicity, surgical recovery, and treatment feasibility. Finally, oncogene-driven disease and real-world populations may require treatment pathways distinct from those derived from molecularly unselected clinical-trial populations. These unresolved questions and corresponding future research priorities are summarized in Figure 2.

### 6.1. Optimizing Postoperative Treatment Duration and Intensity

The optimal duration of perioperative immunotherapy remains undefined. In the neoadjuvant phase, current practice commonly uses 3 to 4 cycles, but the ideal number of cycles has not been prospectively established across different stages and regimens [61,62]. NeoSCORE directly addressed this issue by comparing 2 versus 3 cycles of neoadjuvant sintilimab plus platinum-based chemotherapy in resectable IB–IIIA NSCLC, showing a higher MPR rate with the 3-cycle regimen than with 2 cycles (41.4% vs. 26.9%) [63]. However, whether additional cycles translate into better long-term survival or mainly increase toxicity remains uncertain. The postoperative phase is even less biologically defined: most phase III perioperative regimens are adopted approximately 1 year of adjuvant ICI, largely reflecting trial design rather than validated duration. Emerging evidence suggesting similar outcomes between shorter and longer postoperative immunotherapy schedules challenges this convention, but remains insufficient to change practice [64].

Future studies should therefore move from fixed-duration adjuvant therapy toward response-adapted treatment intensity. Composite biological risk and postoperative treatment feasibility could be used to prospectively evaluate observation, shortened therapy, continuation, or intensification. Component-specific studies such as ADOPT-lung are expected to clarify whether postoperative ICI is necessary after effective neoadjuvant chemoimmunotherapy and surgery [34]. MRD-guided studies and trial designs may further refine this question by assigning postoperative treatment according to molecular relapse risk rather than fixed regimen duration [52,65]. Until such data mature, de-escalation should remain investigational, particularly outside deeply responding and MRD-negative populations.

### 6.2. Optimizing Chemoimmunotherapy and Exploring Novel Combinations

Despite the rapid expansion of perioperative chemoimmunotherapy trials, the optimal ICI–chemotherapy regimen remains undefined. Multiple regimens are now available, but they differ in efficacy signals, safety profiles, treatment duration, and postoperative feasibility. An immediate challenge is to optimize existing chemoimmunotherapy combinations and identify which benefits patients the most. A recent network meta-analysis comparing multiple ICI-plus-chemotherapy strategies suggested that toripalimab plus chemotherapy and nivolumab plus chemotherapy may represent potentially favorable perioperative options: nivolumab offered the most significant OS improvement, whereas toripalimab performed best in both EFS and pCR, albeit at the cost of a relatively higher safety risk [18]. This supports a more nuanced direction for future trials: refining the choice, sequence, and duration of currently available ICI–chemotherapy regimens before uniformly moving toward more intensive or chemotherapy-free approaches.

Chemotherapy remains a cornerstone of current neoadjuvant and perioperative regimens, but it is also a major driver of toxicity. Chemotherapy-free or chemotherapy-minimized strategies therefore remain attractive, particularly for patients at high risk of chemotherapy-related toxicity, yet they should not be assumed to be superior to chemoimmunotherapy. Dual-checkpoint blockade, such as nivolumab plus ipilimumab, has shown activity in resectable NSCLC and may reduce chemotherapy exposure in selected patients; however, the available data are mainly phase II or exploratory, and comparisons have generally been made against chemotherapy rather than established chemoimmunotherapy regimens [66]. Similarly, PD-1/lymphocyte-activation gene 3 (LAG-3) blockade has demonstrated feasibility and preliminary activity before surgery, but its role as a chemotherapy-sparing strategy remains investigational [67]. Thus, chemo-free treatment should be viewed as a biologically rational research direction, not as an intrinsically better approach.

Beyond currently available ICI–chemotherapy platforms, novel combinations are rapidly entering the perioperative space. Strategies involving ICI plus antiangiogenic or multitargeted agents, bispecific antibodies, antibody–drug conjugates, and immune-metabolic modulators are being tested in biomarker-defined populations [68,69,70]. However, these approaches should not distract from the central unresolved question: whether treatment intensification is needed after surgery, and in whom. Future studies should avoid adding agents uniformly and should instead prioritize appropriate treatment among patients with residual pathological or molecular risk.

### 6.3. Driver-Positive Disease

Patients with actionable driver alterations represent a distinct, molecularly stratified perioperative pathway. Comprehensive molecular profiling before initiation of neoadjuvant systemic therapy is therefore important, as the identification of an actionable driver may fundamentally alter treatment selection. In resected EGFR-mutant NSCLC, the ADAURA trial established adjuvant osimertinib as an effective postoperative strategy with significant disease-free and overall survival benefits [71], while the ALINA trial demonstrated a marked disease-free survival benefit with adjuvant alectinib over chemotherapy in resected ALK-positive NSCLC [72]. Targeted therapies are also moving into the neoadjuvant setting. In the phase III NeoADAURA trial, neoadjuvant osimertinib with or without chemotherapy significantly increased MPR compared with chemotherapy alone in resectable EGFR-mutant NSCLC, although event-free survival data remain immature [73].

By contrast, perioperative immunotherapy in driver-positive disease remains exploratory, as these patients were largely excluded from pivotal ICI trials, with only small numbers enrolled in some studies. The NEOTIDE/CTONG2104 trial demonstrated that selected patients with resectable EGFR-mutant NSCLC may achieve pathological responses to neoadjuvant chemoimmunotherapy, but these data are not sufficient to establish routine use [74,75]. Beyond this, exploratory perioperative combinations incorporating ICIs with multitargeted antiangiogenic agents, such as anlotinib plus sintilimab, have also shown preliminary activity in resectable NSCLC [76]. However, as these studies were not specifically conducted in oncogene-driven populations, whether such strategies benefit this subgroup remains unknown and requires dedicated investigation. In the future, more prospective studies are expected to define how genotype-directed therapy should be positioned, sequenced, or selectively integrated with chemotherapy and immunotherapy rather than applying a uniform perioperative ICI strategy across molecular subgroups.

### 6.4. Generalizability and Real-World Implementation

The generalizability of current perioperative evidence remains limited. Most pivotal trials enrolled selected patients with good performance status, adequate organ function, and access to multidisciplinary care. Follow-up remains relatively short for several regimens, limiting assessment of late recurrence, chronic immune toxicity, and post-recurrence treatment effects [77]. Additional limitations include selection bias, underrepresentation of elderly or frail patients, racial and geographic imbalance, heterogeneity in chemotherapy backbones, and exclusion or limited representation of patients with actionable driver alterations [62,78].

Real-world implementation also depends on practical factors that are not fully captured in clinical trials: surgical recovery, adjuvant treatment initiation, completion rates, financial burden, MRD assay availability, and access to experienced thoracic oncology teams. Future studies should therefore incorporate real-world cohorts, patient-reported outcomes, standardized MRD testing, and pragmatic endpoints such as treatment completion, time to surgery, postoperative recovery, and quality of life.

## 7. Conclusions

Perioperative chemoimmunotherapy has reshaped the curative-intent treatment landscape for resectable NSCLC, but current trials do not determine whether continued postoperative immunotherapy is necessary for every patient after neoadjuvant chemoimmunotherapy. Postoperative risk is heterogeneous, and patients achieving pCR with sustained postoperative MRD negativity, favorable baseline biology, and adequate recovery may represent a particularly attractive population for prospective de-escalation studies.

The next step is to move from regimen-defined treatment toward prospectively tested response-guided strategies integrating pathological response and ypN status, ctDNA/MRD, immune contexture, baseline risk, and treatment feasibility. Future studies should clarify optimal adjuvant duration, identify de-escalation and escalation populations, test novel combinations in residual-risk groups, develop molecularly stratified strategies for driver-positive disease, and validate implementation in real-world settings. Ultimately, the goal is not simply to expand perioperative immunotherapy, but to deliver the appropriate postoperative treatment intensity to the appropriate population.

## Figures and Tables

**Table 1:** Landmark neoadjuvant and perioperative chemoimmunotherapy studies in resectable NSCLC.

	N° Pts	ICI Agent	Clinical Stage	Pathological Response	EFS Outcome	HR EFS	OS Outcome	HR OS	Safety Outcome
**Perioperative regimen**									
NADIM II	86	Nivolumab	IIIA	pCR: 36.8% MPR: 52.6%	2-y PFS 67.2% ^1^	0.47 (95% CI 0.25–0.88) ^1^	2-y OS 85%	0.43 (95% CI 0.19–0.98)	Grade ≥ 3 AE 19% ^2^
AEGEAN	802	Durvalumab	IIA–IIIB	pCR: 17.2% MPR: 33.3%	2-y EFS 63.3% 3-y EFS 60.1%	0.69 (95% CI 0.55–0.88)	—	—	Grade ≥ 3 TRAE 32.4% irAE 25.4%
Neotorch	501	Toripalimab	II–III	pCR: 24.8% MPR: 48.5%	2-y EFS 64.7%	0.4 (95% CI 0.28–0.57)	—	0.62 (95% CI 0.38–1.00)	Grade ≥ 3 AE 63.4% irAE 42.1%
KEYNOTE 671	797	Pembrolizumab	II–IIIB	pCR: 18.1% MPR: 30.2%	2-y EFS 62.4% 3-y EFS 54.3% 4-y EFS 48% 5-y EFS 49.9% median EFS 57.1 mo	0.58 (95% CI 0.48–0.69)	2-y OS 80.9% 3-y OS 71.3% 5-y OS 64.6%	0.74 (95% CI 0.59–0.92)	Grade ≥ 3 TRAE 45.2% irAE 26%
Checkmate 77T	461	Nivolumab	IIA–IIIB	pCR: 25.3% MPR: 35.4%	2-y EFS 67% 30-mo EFS 61%	0.61 (95% CI 0.46–0.80)	30-mo OS 78%	0.85 (NS)	Grade ≥ 3 TRAE 32% irAE—
RATIONALE 315	453	Tislelizumab	II–IIIA	pCR: 41% MPR: 56%	2-y EFS 68% 3-y EFS 64.7% 4-y EFS 61.2%	0.58 (95% CI 0.43–0.79)	1-y OS 95% 2-y OS 89% 3-y OS 79.3%	0.65 (95% CI 0.45–0.93)	Grade ≥ 3 TRAE 73% irAE 40.3%
SHR-1316	37	Adebrelimab	II–III	pCR: 29.7% MPR: 51.4%	1-y EFS 77.8%	—	1-y OS 97.3%	—	Grade ≥ 3 TRAE 78.4% irAE—
SAKK 16-14	68	Durvalumab	IIIA(N2)	pCR: 18% MPR: 62%	2-y EFS 67.1% 3-y EFS 53.8% 4-y EFS 49% 5-y EFS 45.9% median EFS 48 mo	—	2-y OS 83% 5-y OS 65.8%	—	Grade ≥ 3 AE 88% irAE—
**Neoadjuvant regimen**									
Checkmate 816	358	Nivolumab	IB–IIIA	pCR: 24% MPR: 36.9%	2-y EFS 63.8% 3-y EFS 57% 4-y EFS 49% 5-y EFS 49.2% median EFS 59.6 mo	0.68 (95% CI 0.51–0.91)	2-y OS 82.7% 3-y OS 78% 5-y OS 65.4%	0.72 (95% CI 0.523–0.998)	Grade ≥ 3 TRAE 33.5% irAE—
NEOSTAR	44	Nivolumab	IB–IIIA	pCR: 18.2% MPR: 32.1%	2-y EFS 73% 3-y EFS 53%	—	2-y OS 91% 3-y OS 86%	—	Grade ≥ 3 TRAE 45% irAE—
phase 1b study of Sintilimab	40	Sintilimab	IA–IIIB	pCR: 16.2% MPR: 40.5%	2-y EFS 72.5% 3-y EFS 70% 5-y EFS 61.5% median EFS 44.4 mo	—	2-y OS 91.7% 3-y OS 86.1% 5-y OS 80.4%	—	Grade ≥ 3 TRAE 10% irAE—
TD-FOREKNOW	94	Camrelizumab	IIIA–IIIB	pCR: 32.6% MPR: 65.1%	2-y EFS 76.9%	0.52 (95% CI 0.21–1.29)	—	—	Grade ≥ 3 TRAE 25.6% irAE 53.5%

Notes: Bold headings indicate the treatment strategy category, distinguishing perioperative regimens from neoadjuvant regimens. Values are presented as reported in the original publications or latest available updates. —, data not available or not reported. Because survival endpoints and safety outcomes were defined and reported differently across trials, these data should be interpreted descriptively rather than compared directly across studies. Median EFS/PFS and median OS are shown when available. ^1^ For NADIM II, EFS was not reported; therefore, PFS was listed in the EFS outcome column, and the reported PFS hazard ratio was listed in the HR EFS column. ^2^ Because grade ≥ 3 TRAEs and irAEs were not reported in NADIM II, grade 3/4 AEs are provided in the safety outcome column. Abbreviations: AE, adverse event; CI, confidence interval; EFS, event-free survival; HR, hazard ratio; ICI, immune checkpoint inhibitor; irAE, immune-related adverse event; MPR, major pathological response; OS, overall survival; pCR, pathological complete response; PFS, progression-free survival; TRAE, treatment-related adverse event; mo, months; NS, not significant.

## Data Availability

No new data were created or analyzed in this study. Data sharing is not applicable to this article.

## Abbreviations

The following abbreviations are used in this manuscript:

ALK	anaplastic lymphoma kinase
APC	article processing charge
BCR	B-cell receptor
CI	confidence interval
CTLA-4	cytotoxic T-lymphocyte-associated protein 4
ctDNA	circulating tumor DNA
DFS	disease-free survival
ECOG PS	Eastern Cooperative Oncology Group performance status
EFS	event-free survival
EGFR	epidermal growth factor receptor
HER2	human epidermal growth factor receptor 2
HR	hazard ratio
HRQoL	health-related quality of life
ICI	immune checkpoint inhibitor
irAE	immune-related adverse event
LAG-3	lymphocyte-activation gene 3
MPR	major pathologic response
MRD	molecular residual disease
NSCLC	non-small cell lung cancer
OS	overall survival
pCR	pathologic complete response
PD-1	programmed cell death protein 1
PD-L1	programmed death-ligand 1
R0	complete resection with no residual tumor
RFS	recurrence-free survival
RVT	residual viable tumor
%RVT	percent residual viable tumor
TLS	tertiary lymphoid structures
TMB	tumor mutational burden
TRAE	treatment-related adverse event
WGS	whole-genome sequencing
ypN	post-neoadjuvant pathological nodal category
